# A room of errors simulation to improve pharmacy operators’ knowledge of cytotoxic drug production

**DOI:** 10.1177/10781552231152145

**Published:** 2023-02-07

**Authors:** Alexandra Garnier, Louise Butaye, Pascal Bonnabry, Lucie Bouchoud

**Affiliations:** 1 Pharmacy Department, 27230 Geneva University Hospitals, Geneva, Switzerland; 2 Institute of Pharmaceutical Sciences of Western Switzerland, School of Pharmaceutical Sciences, 27212 University of Geneva, Geneva, Switzerland; 3 Pharmacy Department, University Clinics of Saint-Luc, Brussels, Belgium

**Keywords:** simulation, knowledge assessment, pharmaceutical training, chemotherapy, errors

## Abstract

**Introduction:**

We used an educational healthcare simulation tool called room of errors (ROE) to raise pharmacy operators’ awareness of potential errors in a chemotherapy production process and assessed its impact on their knowledge and satisfaction.

**Methods:**

Twenty-five errors (compiled from internal procedures, literature and our hospital's reported incidents) were categorised as static (*n* = 7, visible by the participant anytime) and dynamic (*n* = 18, made by a pseudooperator in front of the participant). Our simulated cytotoxic production unit (CPU) hosted the 1 h-simulation. Two pharmacists (supervisor/pseudo-operator) welcomed the trainee for a 10-min briefing. During the 20-min simulation, participants watched the pseudo-operator's gestures in a simulated chemotherapy production process. Participants called out each error observed (recorded by the supervisor). A 20-min debriefing followed. ROE's impact on knowledge was measured through participants’ answers to a before-and after 18-item questionnaire about CPU's procedures and certainty about answers on a scale (0%–100%). Participants evaluated the training using a satisfaction questionnaire (Likert scale, 1–6).

**Results:**

The 14 participants detected 70.4% ± 11.4% of errors. Least-detected errors were “using non-disinfected vials” (42.9%) and “touching syringe plunger” (0%). Critical errors (expired leftovers or glucose instead of sodium chloride) were detected at 57.1%. Knowledge improved from 60.3% to 94.1% (p < 0.001) and certainty from 75.3% to 98.8% (p < 0.001). Participants appreciated this non-judgmental, informative, and original training (satisfaction 95.7%). Some pointed out difficulties settling into the game quickly and visualising static and dynamic errors simultaneously.

**Conclusion:**

This ROE simulation improved operators’ knowledge and certainty. Longer-term testing should be done to measure knowledge retention over time.

## Introduction

Our hospital pharmacy produced 17,000 chemotherapy preparations in 2021. The centralisation of antibody preparation in February 2022 increased this number by 5000 but without any new equipment. Pharmaceutical technology operators are known to face frequent high-stress levels linked to short-staffing^1^ and high levels of interruptions and disturbances.^2^ These factors can lead to errors with significant impacts on patients. A descriptive analysis of centralised chemotherapy preparation in a French hospital reported 140 defective preparations out of 30,819 (0.45%), including major errors such as wrong dose, wrong labelling, unauthorized drugs, incompatible diluents and incompatible bags.^3^ Moreover, good manufacturing practices (GMPs) require the initial and continuous training of operators to improve the quality of cytotoxic preparation.^4^ Continuous training is generally done once a year.^5^ In our hospital, operators undergo an initial three-month training period and are requalified annually by a pharmacy instructor. However, evaluations mainly focus on aseptic manipulation skills and not on the overall competencies required during the entire chemotherapy preparation process.

Simulation is increasingly used to complement traditional teaching approaches such as lectures or distributing theoretical materials.^6–9^ It has become an integral part of medical education at all levels.^10^ Simulation techniques are playful pedagogical tools that can be applied in different ways, such as using error-based simulations (clean rooms containing errors and preparation sheets printed with errors) or game-based simulations (escape games, role-playing games and board games).^6^

The *room of errors* (ROE) concept is particularly interesting because it is a realistic, playful and educational health simulation tool that can be used to raise awareness by discovering errors in a guilt-free context. In an ROE exercise, errors and risks to the patients are deliberately “hidden” in a specially prepared room. Health professionals try, whether individually or in teams, to discover the errors and understand the risks they create. This type of training is already used with health professionals such as physicians,^11^ dentists^12^ and nurses^13^ or for inter-professional purposes between nurses and pharmacists.^14,15^ Considering the advantages of this type of education for improving the quality of operator training, we created a *clean-room of errors* reproducing a cytostatic drug production unit and dedicated to training pharmacy operators and pharmaceutical technology pharmacists.

The present study's primary objective was to raise operators’ awareness of the potential risks and errors existing in the cytotoxic drug preparation process. The second objective was to assess this original training method's impact on operators’ knowledge and their level of satisfaction with the exercise.

## Methods

### Study design

Two pharmacists drew up a table of 25 possible errors in the field of chemotherapy drug production using three different sources: the incidents reported in our hospital relating to the pharmaceutical production unit between April 2019 and August 2021, a non-systematic literature review about *ROE* exercises involving pharmaceutical technology in hospital pharmacies and a list of critical safety points in our existing procedures. These critical safety issues were discussed at a meeting of pharmacists specialising in chemotherapy production. The project was scheduled over six weeks, with one pharmacist working on this project for 80% of their time. Week 1 involved choosing the training method; week 2, choosing the errors and creating the scenario; week 3, adapting our existing simulation room, creating the knowledge questionnaire, testing the training session with an oncology pharmacist to validate the *ROE*'s design; weeks 4–5, performing the *ROE* training sessions; and week 6, analysing the results. The training took place in a simulated chemotherapy preparation unit with a logistics room, a dressing room and a drug preparation room. The equipment used in the drug preparation step included a vertical laminar airflow hood.

**Figure 1.**
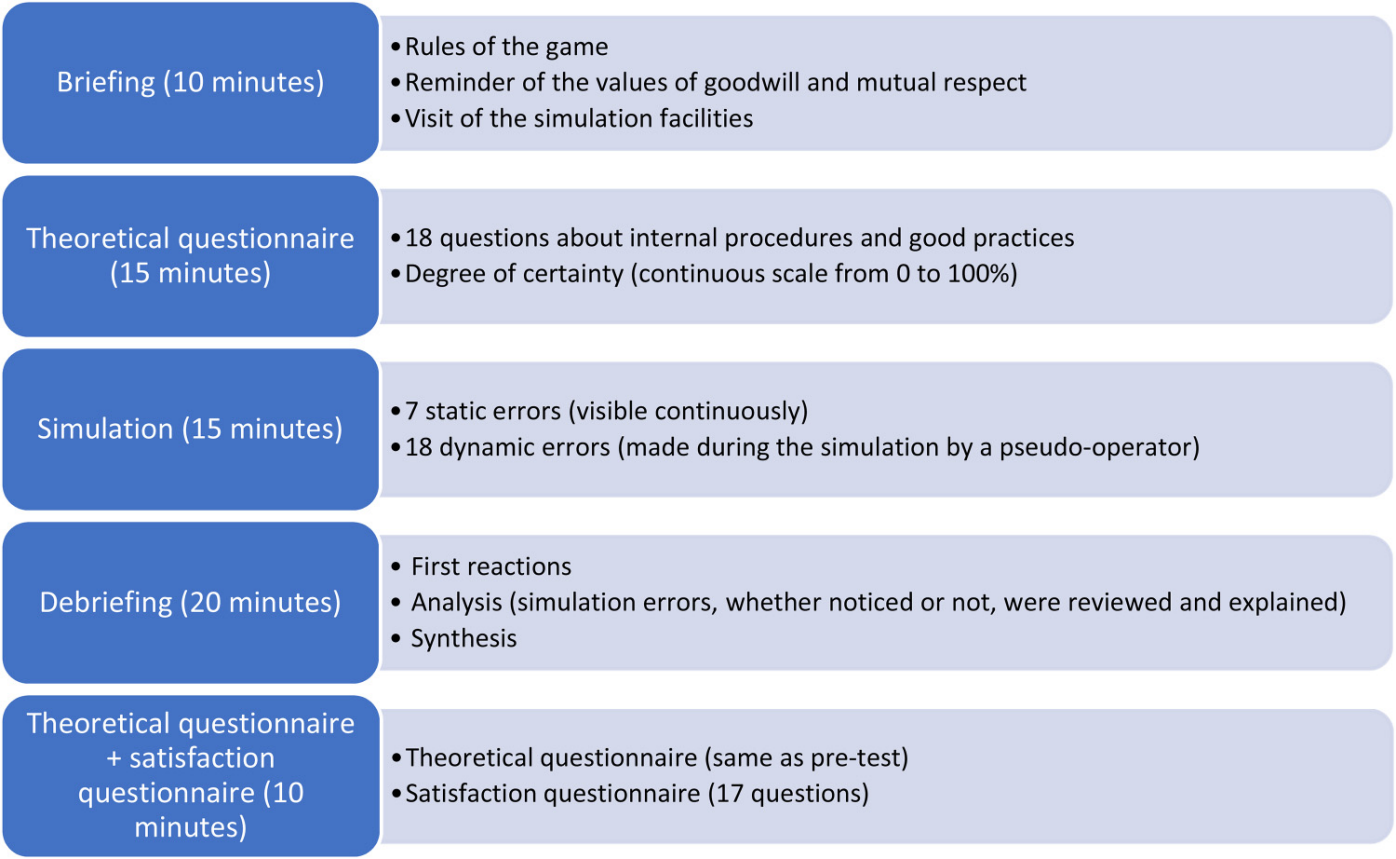
Simulation exercise design.

The training session's organisation followed simulation good practices recommendations (briefing, exercise and debriefing),^16^ which are summarised in Figure 1. Each session involved one operator at a time being welcomed by two pharmacy instructors, one of whom acted as the supervisor, explaining the *room of errors* concept, the rules of the game and reminding the participant of the values of goodwill and mutual respect during the briefing (± 10 min). The other pharmacy instructor was the pseudo-operator, dedicated to playing the role of a pharmacy operator making mistakes. Next, the participant answered the knowledge questionnaire (± 15 min), which was marked by the supervisor while the pseudo-operator gave the participant a guided tour of the simulation facilities. During the simulation exercise itself (± 20 min), 25 errors were made throughout the game in two different forms: static errors that were already present in the room when the participant arrived or dynamic errors made deliberately by the pseudo-operator. Each participant was faced with the same errors. The participant was tasked with observing the room and the pseudo-operator's work and calling out all the errors they saw. The simulation covered every step of a typical chemotherapy preparation process in every zone of the clean room: choice of materials, hand-washing, hand disinfection, dressing, preparation, control and bag shipment.^5^ The game supervisor also stood in the room to record all the errors called out by the participant. Debriefings (± 20 min) followed a standard process of recording participants’ reactions (answers to the questions “How do you feel?” and “Were you able to get into the game?”), analysing their observations (reviewing and explaining all of the errors in the simulation, whether noticed or not) and synthesising final outcomes (answers to the questions “In one sentence, what have you remembered from this exercise?”, “What did you learn?” and “What will you improve in your everyday practice?”). The participant then completed the same theoretical questionnaire as before and a satisfaction survey (± 10 min). In total, the training session lasted approximately 1 h and 15 min.

### Satisfaction

Satisfaction was assessed using Likert scale items rating answers from 0 to 6. Seventeen questions were divided into three categories: training evaluation (seven questions), scenario evaluation (five questions) and personal satisfaction (five questions). The participant's mean result for every category (from 0 to 6) was transformed into a percentage.

### Knowledge improvement

Improvements in knowledge were assessed using the 18 items of a before-and-after questionnaire that looked at various subjects related to the errors in the simulation (Table 1). Our challenge was to respect the concept of pedagogical alignment: assessment tasks should directly address the intended learning outcomes. Regarding the questionnaire, with its 10 multiple choice questions and 8 open-ended questions, we strived to follow the rule of one question per error, as shown in the following three examples. Question no 1 *(“What should we check when assembling the material?”)* was related to the errors of “Wrong diluent bag, wrong vial, wrong syringe in the preparation tray.” Question no 4 (“*What are the hand-washing movements required before entering the clean room?”*) was related to the hand-washing errors, including “The pseudo-operator forgets the interlocked fingers hand-washing step.” Question no 16 *(“Why must the tubing be clamped after purging?”)* was related to the error of “The pseudo-operator does not clamp the preparation after purging.” All the completed questionnaires were marked by the same person. For multiple choice questions with just one correct answer, participants received one point for choosing correctly. For multiple choice questions with several correct answers expected, 1 point was given if all the participant's choices were correct, half a point was given if one or several correct choices were missing and zero points were given if no correct answers were chosen. For the open-ended questions, we used a list of keywords representing the expected answers. One point was given if the participant answered using one or several keywords. Questions answered without using a keyword received zero points. Participants also had to estimate their degree of certainty about each of their answers using a continuous scale from 0% to 100%.^17^

**Table 1.** Theoretical knowledge questionnaire.

No	Question	Type	Answer expected
1	What should you check when assembling the material?	Open-ended	The preparation number The products The solvents heir batch numbers and expiry dates
2	What is the minimum time required for hand washing before entering the clean room (according to the WHO)?	MCQ	20 to 40 s 30 to 50 s **40 to 60 s** 60 to 80 s
3	What is the minimum time required for hand disinfection (according to the WHO)?	MCQ	10 to 20 s **20 to 30 s** 20 to 40 s 40 to 60 s
4	Name the movements for hand-washing before entering the clean room.	Open-ended	Palm against palm Back of the hands Interlocked fingers Back of the fingers in the palm of the hands Thumbs Nails
5	What is the minimum hand-washing procedure before entering a clean room?	MCQ	Simple hand-washing with soap and water **Hygienic hand-washing with soap** Surgical hand-washing with antiseptic soap, in three steps
6	Why should you dry your hands completely by dabbing and not rubbing?	Open-ended	To avoid **spreading residual bacteria**
7	Why use a neutral soap (e.g., Lactacyd) for hand-washing before entering the clean room?	Open-ended	**Reaction** between anionic ordinary soap and cationic (chlorhexidine disinfectant). Lactacyd is a neutral soap.
8	Why not use hydroalcoholic solution to disinfect gloves?	Open-ended	Because it contains an **emollient agent** that can make the gloves **permeable**
9	When should you remove your surgical mask (worn against COVID-19) when entering the clean room?	MCQ	In the changing room **Before washing my hands** I keep it on because I must enter the clean room with a mask
10	What does a vertical laminar airflow hood do?	MCQ	**It protects the operator** **It protects the preparation from external contamination** It makes objects sterile
11	Why must the workspace under a vertical laminar airflow be tidy?	Open-ended	Not to **disturb the airflow direction**
12	How should you clean a vertical laminar airflow hood or the area beneath it?	MCQ	**Using a zig-zag movement** Not using a zig-zag movement From bottom to top **From top to bottom** **From the cleanest area to the dirtiest area** From the dirtiest to the cleanest area **From the back to the front** From the front to the back
13	Out to what distance does an object disturb the airflow?	MCQ	1 time its diameter 2 times its diameter **3 times its diameter** 4 times its diameter
14	How many particles can be emitted by a single unprotected movement of the head or hands?	MCQ	Up to 100,000 particles Up to 200,000 particles **Up to 500,000 particles** Up to 700,000 particles
15	What should you do if your gloves come outside the airflow?	MCQ	Change the gloves **Disinfect gloves with Chlorhexidine dye** Wait outside the flow until the gloves dry before starting work
16	Why must the tubing be clamped after purging?	Open-ended	Risk of contamination when connecting the bag at the bedside
17	For which product(s) should filter tubing be used?	MCQ	Dacarbazine **Paclitaxel** 5-fluorouracil **Pemetrexed**
18	What should be checked when approving a bag for release?	Open-ended	IV-line clamps Inviolability seal cap Type of solvent Yellow opaque bag Trocar inserted to the maximum Choice of IV-line Time of preparation on the label

### Data analysis

Excel^®^ software was used to tabulate the results of the theoretical questionnaires and participants’ error detection. Paired t-tests were used to compare the results from before and after the training session.

## Results

**Figure 2.**
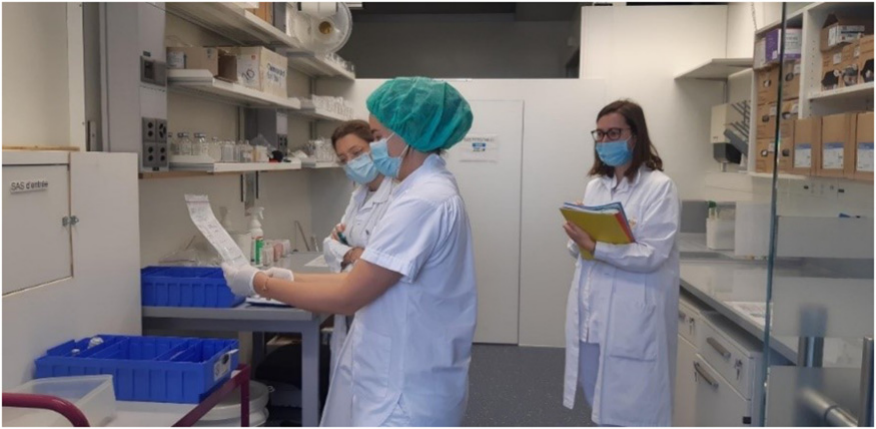
Beginning of the simulation.

**Figure 3.**
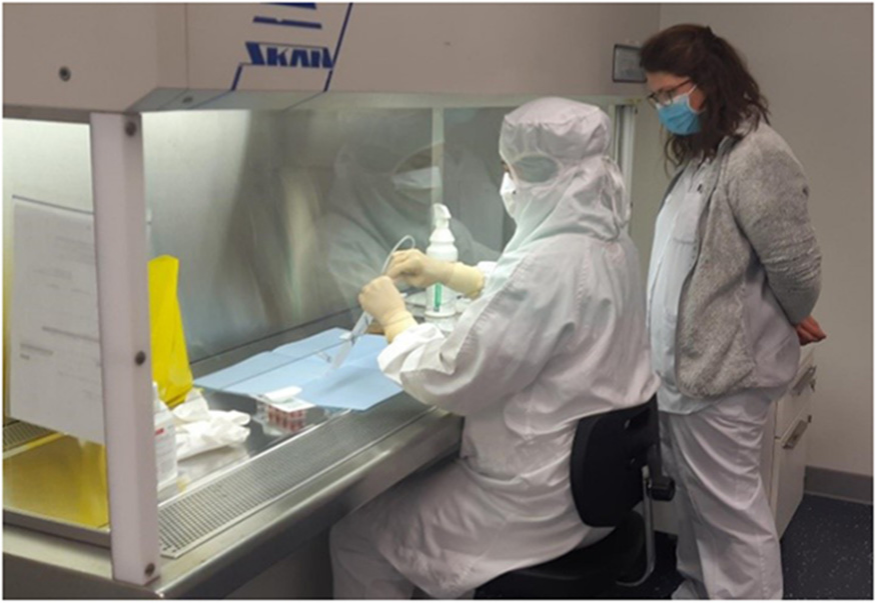
Preparation step during the simulation.

The training sessions took place between October and November 2021 (Figures 2 and 3), and 14 operators participated (4 pharmacists and 10 pharmacy technicians).

### Error detection rate

Errors were separated into the five categories of logistics, hygiene, dressing, preparation and bag approval. Operators found 70.4% ± 11.4% of the errors (Table 2). The most-reported errors were “wrong mask” and “tubing not clamped”, and the least-reported errors were “Vials not disinfected before entering the airflow” and “Touching the syringe plunger”.

**Table 2.** List of errors and results by operator.

Category	No	Error	Operator	1	2	3	4	5	6	7	8	9	10	11	12	13	14	Total (/14)
			Error type															
Logistics	1	Wrong bag (Glucose instead of NaCl 0.9%)	Static	x		x	x		x	x	x		x		x			8
	2	Wrong vial (Treosulfan instead of Etoposide phosphate)	Static	x	x	x	x	x	x		x	x	x	x	x	x	x	13
	3	Wrong syringe (30 mL instead of 10 mL to withdraw 8 mL)	Static		x	x	x	x	x	x	x	x	x	x	x		x	12
Hygiene	4	Hands washed using anionic soap (instead of neutral soap)	Dynamic	x			x			x	x	x	x		x	x	x	9
	5	Hand-washing error (one step of the procedure missed)	Dynamic			x			x	x	x	x	x	x	x		x	9
Dressing	6	Surgical mask not removed before entering the clean room	Dynamic	x	x	x		x	x	x	x	x		x	x	x		11
	7	Wrong mask worn (non-sterile instead of sterile mask)	Dynamic	x	x	x	x	x	x	x	x	x	x	x	x	x	x	14
	8	Sterile gloves not donned correctly	Dynamic			x	x	x	x		x	x	x	x			x	9
	9	Gloves disinfected with the wrong product	Dynamic	x	x	x			x	x		x	x	x	x	x	x	11
	10	Watch is not removed	Dynamic	x	x		x	x	x			x	x	x	x	x	x	11
Preparation	11	There is only one Petri dish in the airflow (instead of two)	Static	x	x	x		x	x	x	x				x	x	x	10
	12	There is clutter inside the airflow (the workspace should be tidy)	Static	x				x	x	x	x	x		x			x	8
	13	The wrong preparation sheet is provided	Static	x	x	x	x	x	x	x	x	x		x	x	x	x	13
	14	The vials are not disinfected before entering the airflow	Dynamic	x					x	x	x			x	x			6
	15	The sterile material is opened outside of the airflow	Dynamic	x		x			x	x		x	x	x	x			8
	16	The gloves are not disinfected inside the airflow	Dynamic	x		x			x	x	x		x		x	x	x	9
	17	The IV line is not clamped	Dynamic	x	x	x	x	x	x	x	x	x	x	x	x	x	x	14
	18	The leftover drugs have expired	Static		x			x	x	x			x		x	x	x	8
	19	The dark part of the needle is touched	Dynamic	/		x			x	x	x				x	x	x	7
	20	The manipulation is done without any pads	Dynamic			x	x	x	x		x	x		x	x	x		9
	21	The syringe plunger is touched	Dynamic	/														0
	22	The preparation is not packed in an opaque bag	Dynamic	x	x		x	x	x	x	x		x	x	x	x		11
Liberation	23	The presence of the tamper-proof cap is not verified	Dynamic	x		x	x	x	x	x	x	x	x	x	x	x	x	13
	24	The preparation is sent at the wrong temperature	Dynamic	x				x	x	x	x	x	x	x	x	x	x	11
	25	The preparation is sent to the wrong ward	Dynamic			x	x	x	x	x	x	x	x		x	x	x	11
Total (/23 for operator 1 and /25 for others)				17	11	17	13	16	23	20	20	17	17	17	22	17	18	

X = error detected; / = error forgotten by the pseudo-operator; No = operator.

### Satisfaction

Mean satisfaction levels with the overall training session and the scenario were 96.5% and 93.5%, respectively. Personal satisfaction about the benefits of this exercise was rated 96.9%. Total average satisfaction was thus 95.7%. (Table 3). Positive comments were given on the learning environment (*“good atmosphere”, “very interesting”, “informative and educational”, “lack of judgement appreciated”, “very intense”*) and the training session's impact (*“questioning one's habits”, “efficient reminder of the procedures”, “a good way to improve oneself*”). Difficulties encountered by the participants concerned the simulated environment *(“complicated to get involved in the scenario”, “environment was not totally real”)* and the game's rules *(“perturbing not to be doing the actions oneself”, “hard to see static errors and dynamic errors at the same time”).*

**Table 3.** Results of the satisfaction survey.

Category assessment	Question	Mean results (%)
Training	This simulation is relevant to my daily professional practice	94.0
	My prior experience and knowledge enabled me to detect errors easily	87.5
	This *room of errors* model is a relevant pedagogical tool for the continuous training of drug production operators	97.0
	The simulation's playful aspect adds more value to reviewing procedures than “traditional” teaching	97.6
	The simulation's total duration was appropriate/adequate	98.8
	The simulation was well organised	100
	The briefing and debriefing were well run	96.4
Overall assessment of the training		**95.9**
Scenario	Errors were varied	96.4
	The choice of errors was relevant	96.4
	The level of difficulty of the errors was relevant to my professional experience	96.4
	The pseudo-operator made the dynamic errors visible enough	84.5
	The scenario reflected reality	94.0
Overall assessment of the scenario		**93.5**
Personal satisfaction	I have benefited from this room of errors training session	96.4
	This experience will help me improve my daily professional practice	92.9
	I will recommend this exercise to colleagues	100
	The room of errors enabled me to update my knowledge	95.2
	There was a good general atmosphere throughout the simulation	100
Overall personal satisfaction		**96.9**
Overall satisfaction		**95.4**

### Knowledge improvement

**Figure 4.**
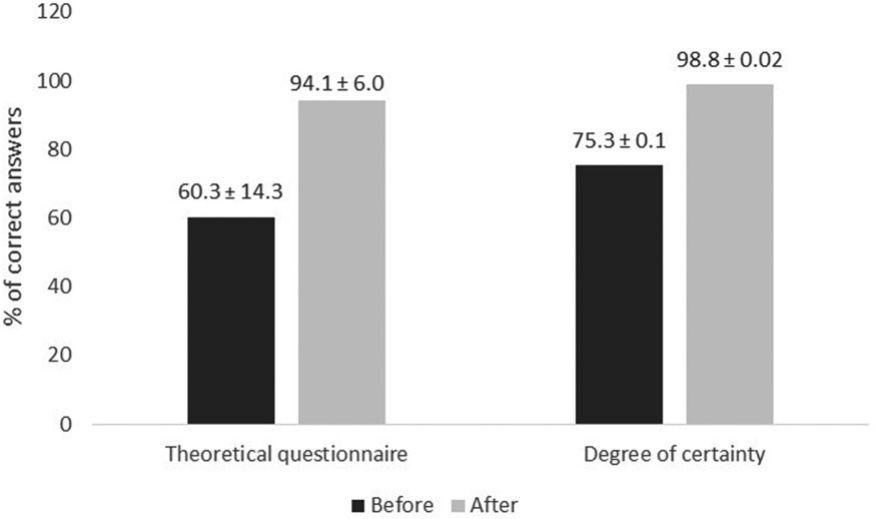
Mean theoretical knowledge questionnaire results and degrees of certainty with their standard deviations (*n* = 14, *p* < 0.001).

Participants’ mean scores for the theoretical questionnaire and their degree of certainty, both before and after training, are provided in Figure 4. There were significant increases in mean scores and degrees of certainty after the simulation exercise (*p* < 0.001).

## Discussion

To the best of our knowledge, this was the first study assessing the efficacy of a *room of errors* simulation for improving knowledge in pharmaceutical technologies.

### Knowledge improvement

According to our non-systematic literature review, *room of errors* simulations of pharmaceutical technology are mainly used to raise operators’ awareness of major potential errors during drug preparation^18,19^ or to enhance their continuous training.^20,21^ These studies only assessed participants’ error detection rates, but there was no focus on improving operators’ knowledge. Hence, our improvements in knowledge scores, from 60.3% to 94.1%, and our improvements in participants’ degrees of certainty, from 75.3% to 98.8%, cannot be directly compared with the pharmaceutical technology literature involving other *room of errors* simulations.

### Error detection rate

The error detection rate reported in the literature on simulation studies in the field of pharmaceutical technology went from 52.2% to 78.5%.^18,22^ Hence, our error detection rate of 70.43% was within the expected range. It is, however, difficult to compare simulation studies that do not contain the same number of errors, categories of errors (logistical, dressing, preparation, etc.) or types of errors (static or dynamic). Loboda *et al*. also used the concept of static and dynamic errors, but they separated them into two separate simulation situations of 20 min each.^23^ Our study's added value was its mixing of static and dynamic errors throughout the same simulation session. Using this approach confronts participants with a situation much closer to reality, where errors can occur at any point during the process.

### Satisfaction

Some negative comments in Loboda *et al*.'s study concerned the lack of immediate feedback after the simulation exercise because of scheduling issues.^23^ Our study rigorously included a personal debriefing of each participant immediately after their simulation training, and they highly appreciated this. The overall mean satisfaction rate in Cotteret *et al*.'s study was 87.1%, with all the respondents being satisfied or very satisfied with the workshop, considering it relevant to their daily work practices and that it provided them with new expertise.^4^ Mean overall satisfaction with our training session was a very encouraging 95.4%, although it should be noted that two item statements received mean ratings under 90%: “My prior experience and knowledge enabled me to detect errors easily” (87.5%) and “The pseudo-operator made the dynamic errors visible enough” (84.5%) (Table 3). Concerning the first statement, all the participating operators had at least five years of experience in chemotherapy preparation in our hospital. This rating may be explained by the choice of errors rarely committed by these participants (touching the needle) or not considered as errors by some of them (removing the needle from the vial without a pad). Concerning the second statement, although each participant was presented with the same static errors, dynamic errors were dependent on the pseudo-operator making them on purpose. Consequently, some unexpected errors made by the pseudo-operator were reported by the participants and some deliberate errors were not reported because they were less visible.

### Design of the *room of errors*

Participants were warned that the simulation would use a laminar airflow hood instead of the isolator used in our production facility, and they were given a guided tour of the clean room before starting the game. However, a few participants admitted in the debriefing that they had been perturbed by the discrepancy between the role-playing situation and their daily professional reality, as in Loboda *et al*.'s study. These issues (lack of fidelity in the equipment used and slight variations in the pseudo-operator's gestures) were also encountered by Loboda *et al*. and may have induced some bias in the results.^23^ This highlights the importance of using a well-rehearsed pseudo-operator and the most realistic equipment.

Evaluating a training session's effectiveness, and therefore its usefulness, is fundamental and should be done by assessing operators’ competencies before and after it. The present study's objective was to improve operators’ knowledge of the chemotherapy preparation process through their ability to detect the errors made by somebody else during this process. The challenge of pedagogical alignment was respected for most of the questions. However, question no 13, *“Out to what distance does an object disturb the airflow?”* was related to the error “There is clutter in the airflow”, and the answer in the standard operating procedures documentation is “3 times its diameter”. It was impossible to answer this question without having read the procedures, and this was only discussed briefly during the debriefing. We encourage future researchers to stick to the rule of one question for one error. Moreover, although using a questionnaire is the easiest and fastest way to collect data, to avoid any memory bias, we recommend that the pre-test and post-test questions be asked in different orders.

### Development of the room of errors

Of five different simulation training tools used in pharmaceutical technology, Bonnet *et al*. considered setting up a *room of errors* to be the most time-consuming.^7^ To put this statement into perspective, every potential error that an operator might make in a clean room has been thought of and implemented in several *room of errors* described in the literature since the first one in 2015.^22^ In the absence of a literature review listing all these errors, it is indeed necessary to take the time to select the mistakes most pertinent to one's working environment and to ready a simulated clean room for this kind of session. We were able to design and execute this study in six weeks, which could be considered quick because one pharmacist dedicated 80% of her working week to this project.

Concerning implementation, our scenario required two trainers for one participant for more than 1 h, which is resource intensive. However, the time spent should be weighed against the efficacy of this kind of active training session. Studies directly comparing the efficacy of different forms of training in the field of pharmaceutical technologies are needed.

### Limitations

The errors reproduced in our simulation were not ostensibly linked to levels of criticality, as in other studies.^18,23,24^ However, as every undetected error was reviewed during the debriefing, the emphasis could be put on serious errors such as a wrong diluent infusion bag or a wrong drug. Encouraging the participants to express their emotions linked to specific errors (the relief of having detected them or regret of having missed them) was an impactful way of highlighting their criticality.

Berthod *et al*. showed that improvements in pharmacy staff's knowledge of GMP were still significant 1 month after an escape room training session.^5^ However, we did not assess medium-term knowledge retention, and it would have been interesting to compare our results with those of Berthod *et al*.

Finally, we made no evaluation of the training session's impact on practice (level 3 in Kirkpatrick's pyramid of learning). Unfortunately, it is not because operators have improved their knowledge that they will necessarily improve their behaviour in their daily practice. A before-and-after evaluation of some simple mistakes would have been interesting, but organising such real-life audits is time-consuming and incompatible with our study schedule.

## Conclusion

The educational, playful learning approach used in our *room of errors* simulation was very much appreciated by our pharmacy production operators. It allowed us to improve their knowledge and their degree of certainty concerning various procedures. Further studies are needed to confirm the efficacy of this kind of training using rigorous evaluation methods that include assessments of the training's impact on operators’ daily practice. Considering the growing number of studies using this training tool in the field of hospital pharmacy chemotherapy production, it would be beneficial for future researchers to have a list of potential errors available and combined with their assessment questions in the form of a user guide. This could be based on the same model as the Patient Safety Switzerland Foundation *ROE* user guide which provides six scenarios that can be set up in any hospital facility.^25^
